# LncRNA SNHG1 acts as a ceRNA for miR-216a-3p to regulate TMBIM6 expression in esophageal squamous cell carcinoma

**DOI:** 10.7150/jca.95127

**Published:** 2024-04-08

**Authors:** Ni Kong, Yuheng Chi, Hong Ma, Dongbo Luo

**Affiliations:** 1 Department of Thoracic Surgery, Xinjiang Medical University Affiliated Tumor Hospital, State Key Laboratory of Pathogenesis, Prevention and Treatment of High Incidence Diseases in Central Asia, Urumqi 830054, China.; 2 Department of Pathology, School of Basic Medical Sciences, Xinjiang Medical University, State Key Laboratory of Pathogenesis, Prevention and Treatment of High Incidence Diseases in Central Asia, Urumqi, Xinjiang 830054, China.; 3 Xinjiang Key Laboratory of Molecular Biology for Endemic Diseases, Xinjiang Medical University, Urumqi, Xinjiang 830017, China.

**Keywords:** ESCC, SNHG1, miR-216a-3p, TMBIM6, ceRNA

## Abstract

**Background:** The long noncoding RNA small nucleolar RNA host gene 1 (SNHG1) has been demonstrated to play a crucial role in the progression of esophageal squamous cell carcinoma (ESCC). The current study aims to explore the deeper molecular mechanisms of SNHG1 in ESCC.

**Methods:** Fifty patients with ESCC were enrolled to assess overall survival. Quantitative real-time PCR was performed to measure the levels of SNHG1, miR-216a-3p, and TMBIM6 in ESCC cells. Functional assessments of SNHG1 on ESCC cells were conducted using CCK-8 assay, flow cytometry, and Transwell assays. Western blot was conducted to detect the protein levels of TMBIM6 and proapoptotic proteins (Calpain and Caspase-12). The interaction among SNHG1, miR-216a-3p, and TMBIM6 was assessed with luciferase reporter assays.

**Results:** Our study revealed that SNHG1 was notably increased in both clinical ESCC samples and cellular lines. Upregulation of SNHG1 in ESCC tissues was indicative of poor overall survival. Functionally, SNHG1 knockdown significantly inhibited the proliferation, migration, and invasion while promoting apoptosis in ESCC cells. Mechanistically, SNHG1 functioned as a competing endogenous RNA by sequestering miR-216a-3p to modulate TMBIM6 levels in ESCC cells. Notably, inhibiting miR-216a-3p or restoring TMBIM6 reversed the inhibitory effect induced by SNHG1 knockdown in ESCC cells.

**Conclusions:** We demonstrate for the first time that SNHG1 may act as a competing endogenous RNA and promote ESCC progression through the miR-216a-3p/TMBIM6 axis. This highlights the potential of SNHG1 as a target for ESCC treatment.

## Introduction

Esophageal cancer is the sixth leading cause of cancer-related mortality globally, with over 400,000 cases reported 1, 2. Esophageal squamous cell carcinoma (ESCC) accounts for more than 90% of all esophageal cancer cases 3. Patients with advanced ESCC patients have poor survival 4, primarily due to recurrence and metastasis 5, 6. Thus, urgent research endeavors are warranted to unveil efficacious diagnostic and therapeutic strategies for ESCC.

Long non-coding RNAs (lncRNAs) are RNA molecules exceeding 200 nucleotides in length 7, 8. They play active roles in diverse cellular and biological processes by orchestrating gene expression through various mechanisms, including epigenetic, transcriptional, and post-transcriptional regulations 9. Additionally, lncRNAs have been implicated significantly in the development of various tumors, including esophageal cancer 10. For instance, lncRNA ATB orchestrates the miR-200b/Kindlin-2 axis to facilitate ESCC progression 11. Similarly, lncRNA RPL34-AS1 regulates ESCC proliferation and migration through the miR-575/ACAA2 axis 12. Furthermore, LINC00680 functions as an oncogene in ESCC development by upregulating CDK6 expression and can promote ESCC progression through miR-497 sponging 13. These findings suggest that lncRNAs could represent promising targets for the diagnosis and treatment of ESCC.

LncRNA SNHG1 has been documented to promote the proliferation, invasion, and metastasis of various tumors, including colorectal cancer, liver cancer, gastric cancer, and lung cancer 14-16. Recent studies have indicated that the aberrant expression of SNHG1 actively contributes to the progression of ESCC 17-19. However, the precise mechanism of SNHG1 in ESCC remains largely elusive.

The competing endogenous RNA (ceRNA) may mediate the gene regulation by lncRNAs 20, where a subset of RNA transcripts reduces the availability of microRNAs (miRNAs) directed towards messenger RNAs (mRNAs) by competitively binding to shared miRNAs 21, 22. For example, LINC00665 enhances the proliferation and induces the epithelial-mesenchymal transition of breast cancer through sequestering miR-379-5p to regulate LIN28B expression 23. Similarly, MATN1-AS1 is aberrantly expressed in glioma and acts as an oncogene by sponging miR-200b/c/429, thereby upregulating CHD1 expression and promoting glioma development 24. Serving as a ceRNA for miR-101-3p, SPRY4‐IT1 promotes the proliferation and metastasis of bladder cancer by regulating EZH2 expression 25.

To investigate the potential involvement of SNHG1 in the ceRNA network in ESCC, we performed predictions utilizing online tools such as Tarbase (http://www.tarbase.com), miRWalk (http://mirwalk.umm.uni-heidelberg.de/) and Targetscan (https://www.targetscan.org). The predictions suggested potential complementary sequences between SNHG1 and TMBIM6 with miR-216a-3p, prompting the hypothesis of a ceRNA network comprising SNHG1/miR-216a-3p/TMBIM6. Based on this, we aim to examine the role of SNHG1 in ESCC and determine whether it influences ESCC development by modulating the miR-216a-3p/TMBIM6 axis.

## Materials and Methods

### Clinical samples

Fifty samples of human ESCC tissues and 30 samples of normal adjacent tissues were obtained from 50 patients diagnosed with ESCC at the Tumor Hospital Affiliated with Xinjiang Medical University. Inclusion criteria for patients with ESCC included: 1) patients with an ESCC diagnosis confirmed through preoperative methods such as CT scan, endoscopy, and pathological biopsy; 2) patients without distant metastases in the liver, lungs, or abdominal cavity as determined by preoperative chest CT, abdominal ultrasound, and other relevant examinations; 3) patients exhibiting normal function of heart, liver, kidney, and other organs, as well as normal blood routine, urine routine, and stool routine tests; and (4) patients who did not receive preoperative treatments such as radiotherapy or chemotherapy. Exclusion criteria for patients with ESCC were: 1) patients with a history of neoadjuvant radiotherapy before surgery; 2) patients who underwent non-radical procedures including palliative surgery, diversion, and exploratory surgery; and 3) patients with severe cardiac, hepatic, pulmonary, or renal comorbidities. All tissues collected were stored at a temperature of -80°C. The work was conducted under the approval of the Ethical Committee of Affiliated Tumor Hospital of Xinjiang Medical University (approval number: K-2023053).

### Cell culture

ESCC cell lines TE-1 and KYSE-150 were purchased from iCellbio (Shanghai, China), and KYSE-30 was obtained from Keycell (Wuhan, China). The cells were maintained in RPMI 1640 medium (Keycell, Wuhan, China), supplemented with 10% fetal bovine serum (ExCell Bio, Jiangsu, China), 100 U/ml penicillin, and 100 μg/ml streptomycin (Keycell, Wuhan, China), at 37°C in a humidified incubator with 5% CO_2_.

### Transfection of cells

The lncRNA Smart Silencer (RiboBio, Guangdong, China), comprising an equal mix of three small interfering RNAs (siRNAs) and three antisense oligonucleotides (ASOs), was transfected into ESCC cells at an optimal final concentration of 100 nM to inhibit the target genes. The transfection in the TE-1 and KYSE-150 cells was carried out using Lipofectamine 2000 (Invitrogen, Shanghai, China) according to the manufacturer's protocol. The sequences of siRNAs and ASOs were as follows: si-SNHG1#1: CCA GCA TCT CAT AAT CTA T; si-SNHG1#2: GTG AAG GAA TGG GAC AAG AC; si-SNHG1#3: CCC TTG AGG ACT GGC TGT CA; ASO#1: AGC TGA GAG GTA CTA CTA AC; ASO#2: GAG GAC ATC AGA AGG TGA A; ASO#3: GCC AGC ACC TTC TCT CTA A. The siRNA negative control (NC), ASO control, and Smart Silencer control were also purchased from RiboBio (Guangdong, China).

### Quantitative real-time PCR (qRT-PCR)

Total RNA was extracted from ESCC tissue samples and transfected cells using an Ambion total RNA extraction reagent (Shanghai, China). The cDNA was obtained according to the protocol of HiScript II (Vazyme, Nanjing, China). The qRT-PCR was conducted on an ABI 7500 instrument (Thermo Fisher, Zhejiang, China) using the SYBR Green mix (Thermo Fisher, Zhejiang, China). The primer sequences for SNHG1, miR-216a-3p, and TMBIM6 are presented in Table 1.

### CCK-8 assay

The cell proliferation was evaluated at various time points (0 h, 24 h, 48 h, 72 h, 96 h, and 120 h) using the CCK-8 assay kit (Keycell, Wuhan, China). Each well was treated with 10 μL of CCK-8 solution and incubated at 37°C for 3 h. Finally, a microplate reader (BioTek, USA) recorded the optical density at 450 nm.

### Flow cytometry analysis

Cell apoptosis was examined by flow cytometry. TE-1 and KYSE-150 cells were collected, resuspended in 500 μL of binding buffer, and stained with 5 μL of Annexin V-FITC/PI (Keycell, Wuhan, China). The reaction was carried out for 5-15 min at room temperature under light shielding.

### Transwell assay

Transwell assays were used to detect cell migration and invasive ability. In the migration assay, TE-1 and KYSE-150 cells (2 × 10^5^/well) were suspended in serum-free RPMI-1640 medium and seeded into the top chamber. In the invasion assay, 2 × 10^5^ cells were seeded into the top chamber pre-coated with Matrigel (Corning, USA). The lower chamber was supplemented with 800 μl of RPMI-1640 containing 10% FBS. Following a 24 h incubation period, the cells in the lower chambers were stained with 0.1% crystal violet. The migrated and invaded cells were enumerated under the microscope (Nikon, Ta2-FL, Japan).

### Luciferase reporter assay

Wild-type (WT) luciferase reporter plasmids (SNHG1-WT and TMBIM6-WT) were constructed by integrating the sequences of SNHG1 or TMBIM6 3ʹUTR, encompassing miR-216a-3p binding sites, into the pYr-MirTarget vectors (yrbio, China). Mutant (MUT) luciferase reporter plasmids (SNHG1-MUT and TMBIM6-MUT) were also synthesized by introducing mutations into the putative binding sites. Subsequently, 293T cells were co-transfected with the constructed luciferase reporter plasmids and either miR-216a-3p or an NC. Following 72 h of transfection, the relative luciferase activity was quantified using the luciferase reporter assay system (Beyotime, China).

### Western blot

The total proteins were extracted from KYSE-150 and TE-1 cells using RIPA buffer containing 1% PMSF, and their concentration was determined using the BCA Protein Assay Kit (G3522, GBCBIO, Guangzhou, China). Subsequently, the proteins underwent separation via SDS-PAGE and were then transferred onto PVDF membranes (Millipore, Billerica, MA, USA). The membrane was incubated in 5% skim milk for 2 h for blocking. GAPDH (AB-P-R001, Goodhere, China) protein served as an internal control. Primary antibodies included Calpain (Bsm-51590m Bioss, China), Caspase-12 (Bs-1105R, Bioss, China), and TMBIM6 (26782-1-AP, Proteintech, China). The incubation with the primary antibody was performed overnight at 4°C. Following this, HRP-conjugated sheep anti-rabbit secondary antibody (A0208, Beyotime, China) and HRP-conjugated sheep anti-mouse secondary antibody (SA00001-1, Proteintech, China) were added and incubated for 2 h.

### Statistical analysis

All data are presented as mean ± standard deviation (SD). Differences were assessed using Student's t-test or one-way analysis of variance. A P-value below 0.05 signifies statistical significance.

## Results

### SNHG1 expression is elevated in ESCC tissues and cells

**Figure 1:**
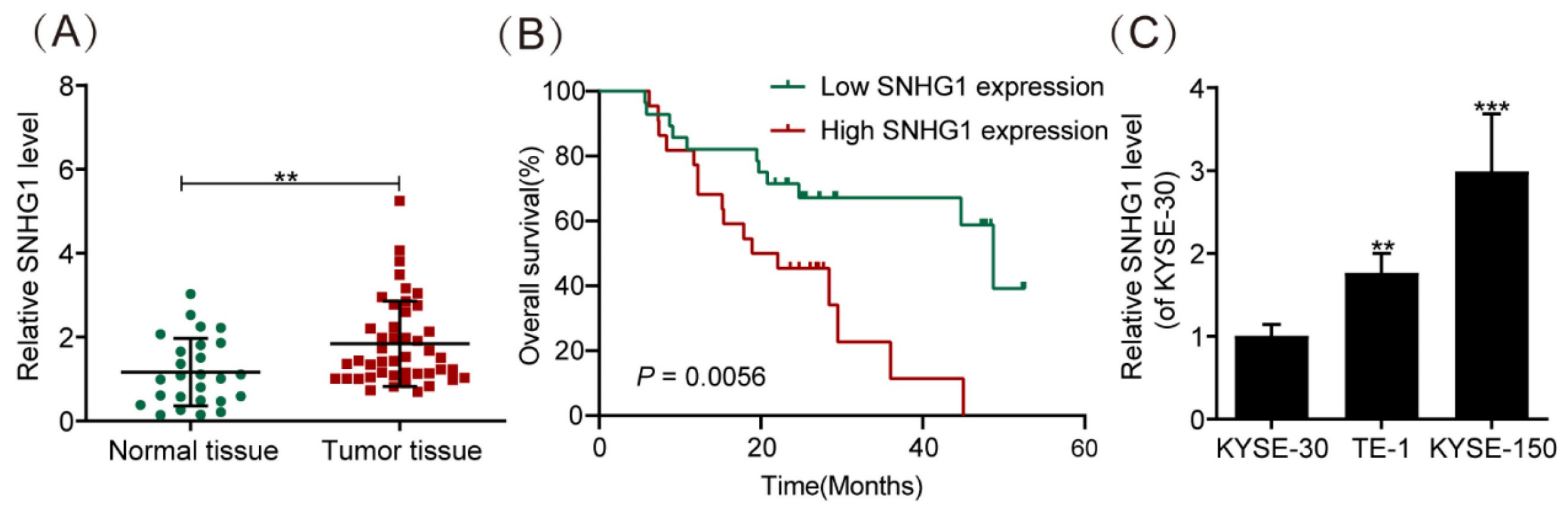
SNHG1 is highly expressed in ESCC tissues and cell lines. (A) qRT-PCR analysis of SNHG1 expression levels in ESCC tissues obtained from surgically treated patients. (B) Kaplan-Meier analysis of the overall survival in a cohort of 50 ESCC patients with high and low SNHG1 expression. The threshold value to determine high and low SNHG1 expression was 1.24. (C) qRT-PCR analysis of SNHG1 expression levels in KYSE-30, TE-1, and KYSE-150 cells. *P < 0.05, **P < 0.01, ***P < 0.001.

We evaluated the abundance of SNHG1 in 30 normal ESCC tissues and 50 ESCC tissues by using qRT-PCR. The results demonstrated significantly higher SNHG1 expression in ESCC tissues compared to normal tissues (Fig. 1A). In addition, the relationship between SNHG1 level and clinicopathological features of ESCC patients was investigated (Table 2). Based on the average value (1.24) of SNHG1 determined by qRT-PCR, we categorized 50 ESCC patients into an SNHG1 high-expression group (n = 22) and an SNHG1 low-expression group (n = 28). Our analysis indicated significant associations between SNHG1 levels and lymph node metastasis (*P* = 0.035), depth of invasion (*P* = 0.026), histological subtypes (*P* = 0.047), and TNM stage (*P* = 0.027). However, no significant associations were found between SNHG1 levels and age, tumor size, gender, or tumor status. Furthermore, Kaplan-Meier analysis revealed that ESCC patients with elevated levels of SNHG1 generally experienced poorer overall survival compared to those with lower levels of SNHG1 (Fig. 1B), suggesting the potential of SNHG1 as a prognostic biomarker for ESCC. In our previous study, we found that the expression of SNHG1 in ESCC cell lines TE-1, Eca-109, KYSE-170, and KYSE-150 was higher than in human oesophageal epithelial (HET-1) cells 26. For further validation, we assessed SNHG1 levels in ESCC cell lines (KYSE-30, TE-1, and KYSE-150) by using qRT-PCR. The results revealed significantly elevated levels of SNHG1 in TE-1 and KYSE-150 cells compared to KYSE-30 cells (Fig. 1C).

### SNHG1 enhances the malignant behaviors of ESCC cells

**Figure 2:**
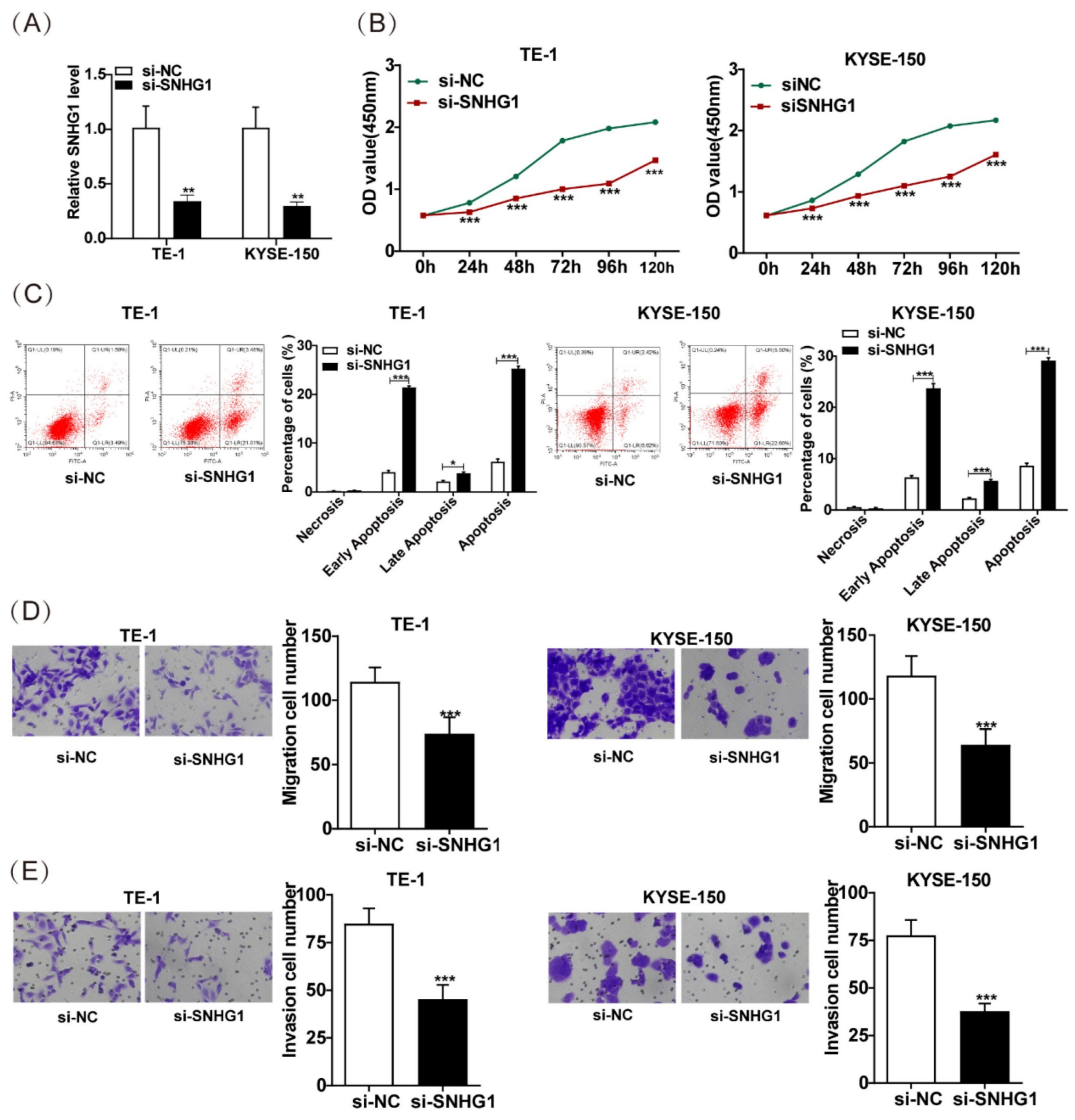
SNHG1 amplifies the malignant tendencies in ESCC cells. (A) qRT-PCR analysis of the expression of SNHG1 in TE-1 and KYSE-150 cells after transfecting si-SNHG1 or si-NC. (B) Cell proliferation capacities were assessed using CCK-8 assays at various time intervals (0 h, 24 h, 48 h, 72 h, 96 h, and 120 h). (C) Apoptosis rates were determined through flow cytometry analysis. (D and E) The migration and invasion abilities of the cell were evaluated via Transwell assays. *P < 0.05, **P < 0.01, ***P < 0.001.

To explore the role of SNHG1 in ESCC, we further knocked down SNHG1 in TE-1 and KYSE-150 cells, which exhibited relatively higher levels of SNHG1. Subsequent qRT-PCR analysis indicated a notable decrease in SNHG1 abundance in TE-1 and KYSE-150 cells following si-SNHG1 transfection compared to the siRNA NC group (Fig. 2A). The CCK-8 assay demonstrated substantial inhibition of cell proliferation in TE-1 and KYSE-150 cells after SNHG1 knockdown (Fig. 2B). Flow cytometry revealed that SNHG1 knockdown significantly increased the apoptosis rate in TE-1 and KYSE-150 cells (Fig. 2C). Additionally, Transwell assays showed a marked reduction in the migration and invasion abilities of TE-1 and KYSE-150 cells after SNHG1 silencing (Figs. 2D and 2E). These results suggest that high expression of SNHG1 in ESCC cells accelerates ESCC progression, highlighting SNHG1 as an oncogenic lncRNA in ESCC development.

### SNHG1 sponges miR-216a-3p

**Figure 3:**
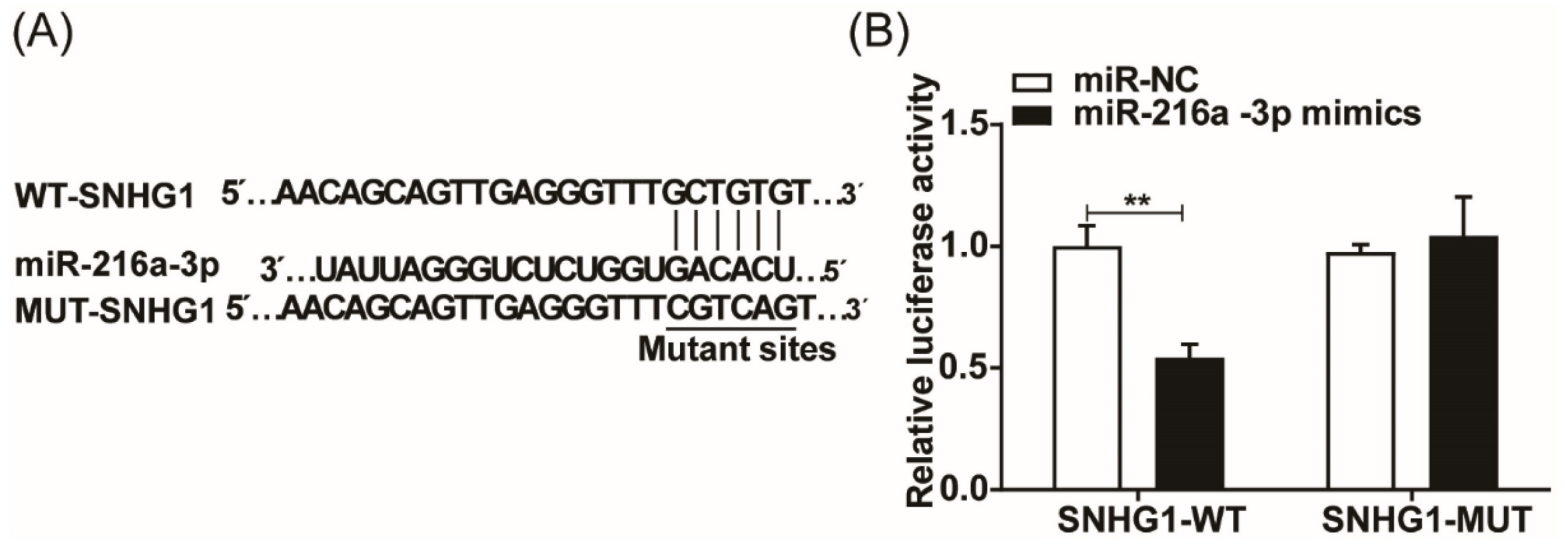
SNHG1 sponges miR-216a-3p. (A) The binding sites of SNHG1 and miR-216a-3p were predicted using the Tarbase database. (B) Luciferase reporter assay verified the specific binding between SNHG1 and miR-216a-3p. **P < 0.01.

To elucidate the underlying mechanism of SNHG1 in ESCC development, we predicted the binding sites between miR-216a-3p and SNHG1 using the TarBase database (Fig. 3A). To verify the potential interaction between SNHG1 and miR-216a-3p, a dual luciferase reporter assay was conducted. The results indicated that in 293T cells transfected with miR-216a-3p mimics, the luciferase activity of SNHG1-WT was significantly diminished, while that of SNHG1-MUT remained unaltered (Fig. 3B). These findings strongly suggest that miR-216a-3p targets SNHG1.

### miR-216a-3p inhibitor weakens the inhibitory effect of SNHG1 silencing on the malignant behaviors in ESCC cells

**Figure 4:**
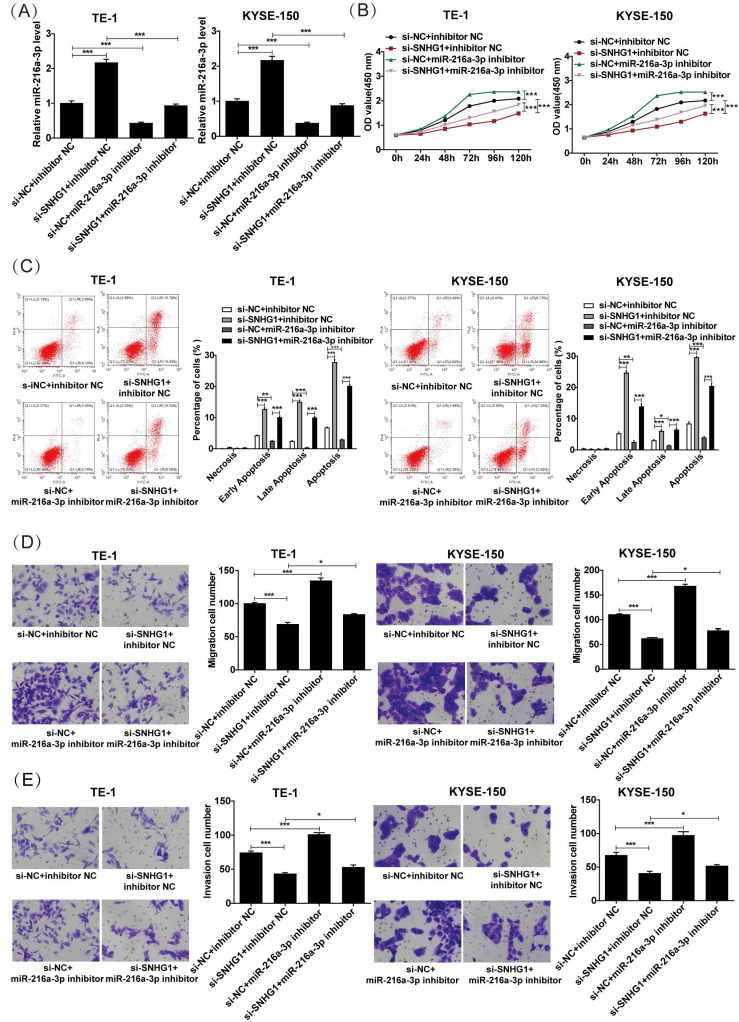
The proliferation, apoptosis, migration, and invasion of ESCC cells after co-transfection of the miR-216a-3p inhibitor and si-SNHG1. (A) qRT-PCR analysis of the level of miR-216a-3p in TE-1 and KYSE-150 cells after transfection of si-NC+inhibitor NC, si-SNHG1+inhibitor NC, si-NC+miR-216a-3p inhibitor and si-SNHG1+ miR-216a-3p inhibitor. Cell proliferation (B), apoptosis (C), migration (D), and invasion (E) of TE-1 and KYSE-150 cells were assessed by CCK-8, flow cytometry, and Transwell assays. *P < 0.05, **P < 0.01, ***P < 0.001.

The proliferation, apoptosis, migration, and invasion of ESCC cells were investigated after co-transfection of miR-216a-3p inhibitor and si-SNHG. As depicted in Fig. 4A, co-transfection with si-SNHG1 and the miR-216a-3p inhibitor in both TE-1 and KYSE-150 cells markedly reduced the expression of miR-216a-3p compared to cells transfected with si-SNHG1 only. CCK-8 assay revealed that si-SNHG1 transfection suppressed cell proliferation in TE-1 and KYSE-150 cells, while co-transfection with the miR-216a-3p inhibitor effectively reversed this effect (Fig. 4B). As shown in Fig. 4C, co-transfection with the miR-216a-3p inhibitor significantly counteracted the promoting effect of si-SNHG1 on cell apoptosis in TE-1 and KYSE-150 cells. Furthermore, the Transwell assay demonstrated that the inhibitory effects of si-SNHG1 on cell migration and invasion were reversed upon transfection with the miR-216a-3p inhibitor (Figs. 4D and 4E). Additionally, independent inhibition of miR-216a-3p enhanced the proliferation, migration, and invasion capabilities of TE-1 and KYSE-150 cells while suppressing apoptosis of the cells. These results demonstrated that the suppressive effect of si-SNHG1 on ESCC cells could be reversed by miR-216a-3p inhibition, indicating that the effects of SNHG1 on ESCC cells may be associated with miR-216a-3p.

### SNHG1 regulates TMBIM6 by sponging miR-216a-3p in ESCC cells

**Figure 5:**
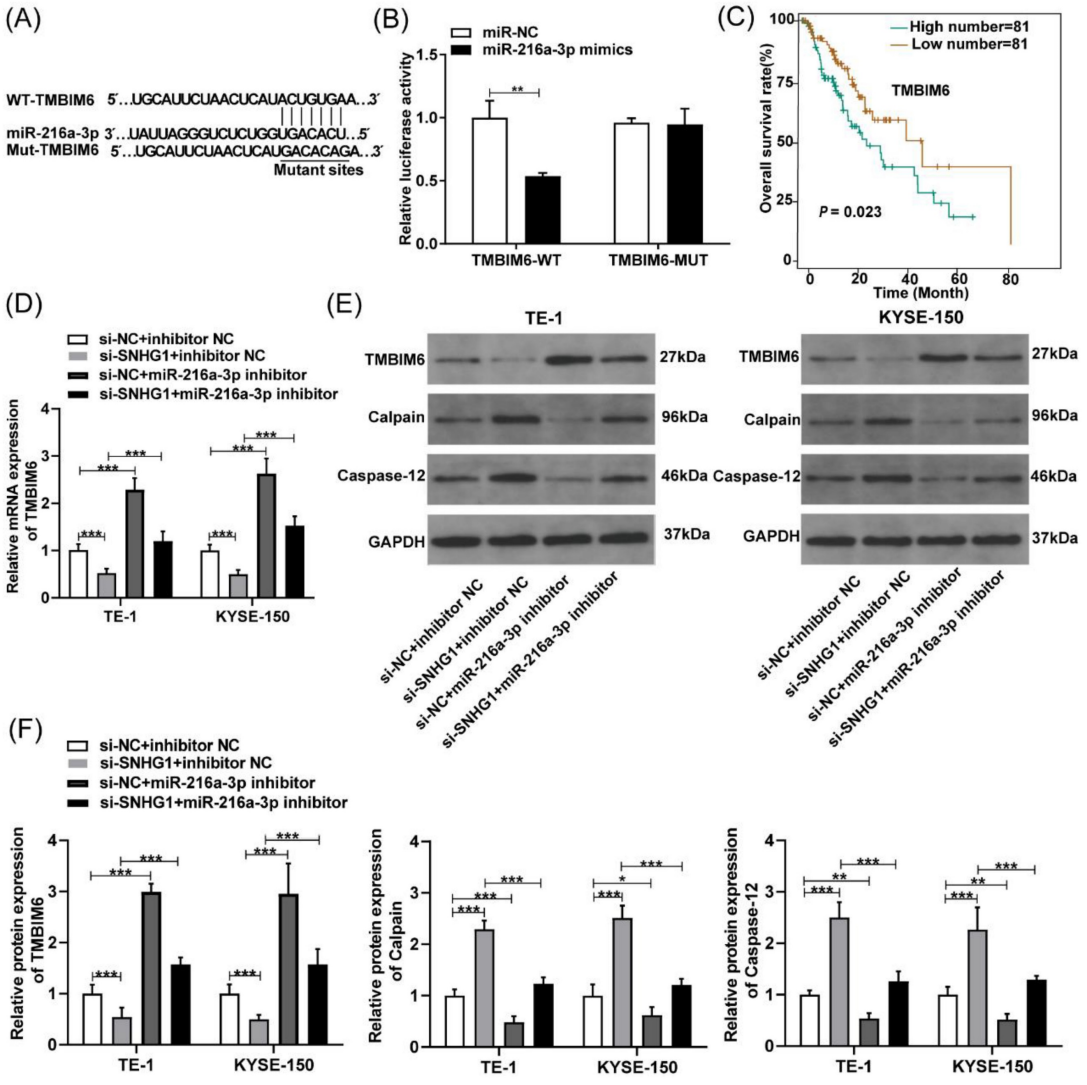
SNHG1 regulates TMBIM6 by sponging miR-216a-3p. (A) The binding sites of miR-216a-3p and TMBIM6 were predicted using the TargetScan database. (B) Luciferase reporter assay was performed to ascertain the interaction between miR-216a-3p and TMBIM6. (C) Survival analysis was performed in a cohort of 162 esophageal cancer patients (n = 81 with low expression, n = 81 with high expression) using data from the TCGA database, revealing a significant association (P = 0.023, P < 0.05) between TMBIM6 levels and esophageal carcinoma prognosis. (D) The mRNA levels of TMBIM6 were detected using qRT-PCR. (E) and (F) The TMBIM6, Calpain, and Caspase-12 protein expression changes were analyzed by Western blotting. *P < 0.05, **P < 0.01, ***P < 0.001.

To further determine the possible downstream genes affected by miR-216a-3p in ESCC, we analyzed the putative binding sites of miR-216a-3p with TMBIM6 using Targetscan (Fig. 5A). Subsequently, a luciferase reporter assay was employed to validate this interaction, which revealed that the miR-216a-3p mimics significantly attenuated the luciferase activity of the TMBIM6-WT group, while no significant change was observed in the luciferase activity of the TMBIM6-MUT group (Fig. 5B). TMBIM6 is associated with enhanced migration, invasion, and poor prognosis in various malignancies 27. Through analysis of the TCGA database, we initially observed a significant association between high TMBIM6 expression and poor prognosis in esophageal cancer patients (Fig. 5C). Moreover, qRT-PCR results indicated that TMBIM6 expression was downregulated after SNHG1 knockdown and upregulated after miR-216a-3p inhibition in TE-1 and KYSE-150 cells (Fig. 5D). Additionally, qRT-PCR results revealed a significant reduction in the expression of TMBIM6 in ESCC cells co-transfected with the miR-216a-3p inhibitor compared to ESCC cells transfected only with si-SNHG1 (Fig. 5D). Western blotting results suggested that inhibition of miR-216a-3p led to the upregulation of TMBIM6 protein expression and downregulation of pro-apoptotic proteins Calpain and Caspase-12 (Figs. 5E and 5F). Knockdown of SNHG1 resulted in a decrease in TMBIM6 protein expression and an increase in protein levels of Calpain and Caspase-12, while this trend was markedly reversed after co-transfection with the miR-216a-3p inhibitor (Figs. 5E and 5F). Moreover, inhibiting miR-216a-3p alone enhanced the mRNA and protein expression of TMBIM6 while concurrently inhibiting the protein expression of Calpain and Caspase-12 (Figs. 5D-5F). Therefore, the competitive binding of SNHG1 to miR-216a-3p could lead to the upregulation of TMBIM6 expression and the inhibition of pro-apoptotic proteins Calpain and Caspase-12 in ESCC cells.

### SNHG1 exerts oncogenic effects in ESCC by modulating the miR-216a-3p/TMBIM6 axis

**Figure 6:**
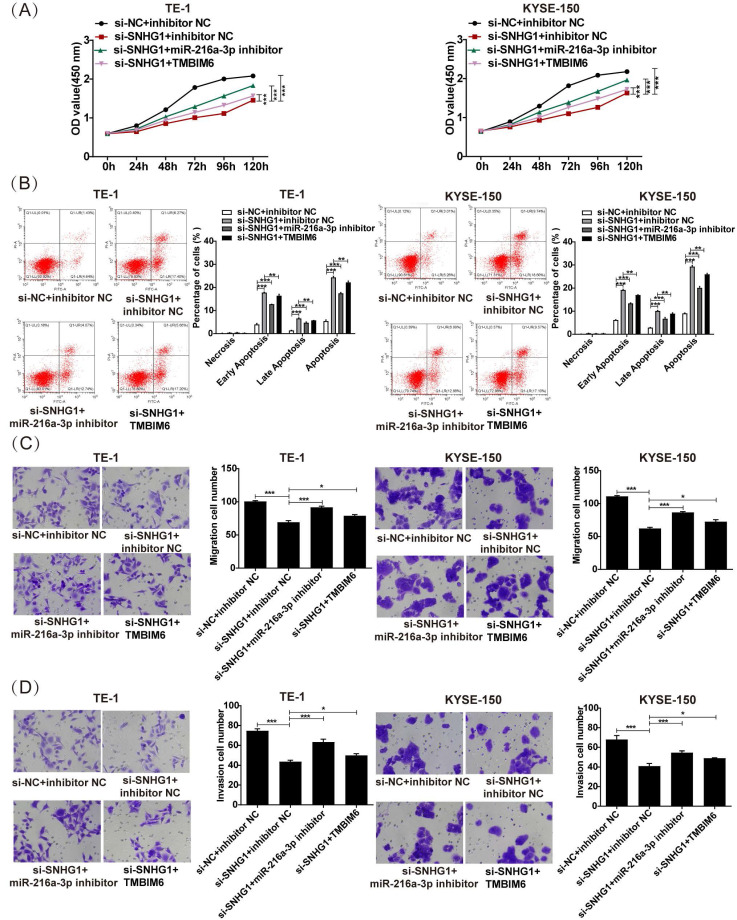
SNHG1 exerts its tumorigenic function in ESCC by regulating the miR-216a-3p/TMBIM6 axis. (A) Cell proliferation, (B) apoptosis, (C) migration, and (D) invasion were detected in TE-1 and KYSE-150 cells following transfection with si-NC+inhibitor NC, si-SNHG1+ inhibitor NC, si-SNHG1+ miR-216a-3p inhibitor, si-SNHG1+ TMBIM6 by CCK-8, flow cytometry, and transwell assays. *P < 0.05, **P < 0.01, ***P < 0.001.

To clarify the specific impact of the SNHG1/miR-216a-3p/TMBIM6 axis on ESCC cellular activities, we conducted rescue assays in TE-1 and KYSE-150 cells. As depicted in Fig. 6A, the reduction in proliferation induced by si-SNHG1 in TE-1 and KYSE-150 cells was rescued by miR-216a-3p suppression or TMBIM6 overexpression. Furthermore, the apoptosis of TE-1 and KYSE-150 cells triggered by si-SNHG1 was significantly reversed by miR-216a-3p inhibition or TMBIM6 overexpression (Fig. 6B). Lastly, inhibition of miR-216a-3p or overexpression of TMBIM6 mitigated the inhibition of cell migration and invasion mediated by si-SNHG1 in TE-1 and KYSE-150 cells (Figs. 6C and 6D). These findings collectively imply that SNHG1 promotes malignant behaviors in ESCC by modulating the miR-216a-3p/TMBIM6 axis.

## Discussion

Many studies have elucidated the significant involvement of lncRNAs in the occurrence and development of multiple cancers 11, 28, 29. Extensive research endeavors have been devoted to elucidating the effects and underlying mechanisms of lncRNAs, aiming to identify promising therapeutic approaches and clinical prognostic markers for tumors 30, 31.

The present study seeks to unveil the precise role of SNHG1 in ESCC. Aberrant SNHG1 expression has been linked to the biological processes and regulatory mechanisms of numerous cancers 14, 32. For instance, SNHG1 promotes non-small cell lung carcinoma progression via targeting miR-101-3p 33. In prostate cancer cells, SNHG1 competitively inhibits miR-377-3p expression, leading to AKT2 hyperactivation, which facilitates proliferation and suppresses apoptosis in prostate cancer cells 34. Moreover, SNHG1 enhances HK2 expression by competitively regulating miR-143-3p expression, thus promoting bladder cancer cell proliferation 35. Several studies have underscored the significance of SNHG1 in the development of ESCC. For instance, SNHG1 acts as an undegradable sponge for the tumor suppressor miR-338 within esophageal cancer cells, thus promoting cell proliferation 17. Furthermore, the upregulation of SNHG1 has been shown to enhance cell proliferation and invasion by modulating the Notch signaling pathway in ESCC 18. Additionally, the progression of ESCC is regulated by the SNHG1 via the miR-204/HOXC8 axis 19. Our previous research found that miRNA-21 promoted cell proliferation in ESCC by upregulating the expression of lncRNA SNHG1 26.

These findings imply that SNHG1 serves as an oncogenic lncRNA contributing to the initiation and progression of ESCC. Nonetheless, the effects of SNHG1 on the migration, invasion, and apoptosis of ESCC cells, as well as the underlying mechanisms, remain inadequately investigated. Here, we observed that SNHG1 was notably upregulated in ESCC tissues, which was strongly associated with poor prognosis. Subsequent functional experiments illustrated that SNHG1 knockdown suppressed the proliferative, migratory, and invasive capabilities of ESCC cells while inducing apoptosis in ESCC cells. Therefore, downregulation of SNHG1 expression may represent a promising target for targeted therapy in ESCC.

LncRNAs can sequester miRNAs and impede their repression of target genes 22. SNHG1 has been reported to interact with various miRNAs, such as miR-195-5P 36, miR-377-3p 37, miR-129-3p 38, and miR-181b-5p 39. It is suggested that SNHG1 can regulate the activity of Parkinson's disease model cells by affecting miR-216a-3p 40. In this study, Tarbase analysis and luciferase reporter assay verified that miR-216a-3p was a pivotal target of SNHG1. Notably, miR-216a-3p has been reported to exert inhibitory effects in various tumors. In lung cancer, miR-216a-3p suppresses the proliferation and enhances the apoptosis of A549 cells 41. In colon cancer, miR-216a-3p inhibits CRC cell proliferation by regulating the expression of COX-2 and ALOX5 42. In cervical cancer, overexpression of miR-216a-3p significantly inhibits the proliferation and invasiveness of Ca_Ski and SiHa cells 43. However, the role of miR-216a-3p in ESCC remains unclear. Here, we found that SNHG1 served as a negative regulator of miR-216a-3p, as inhibition of SNHG1 caused elevated expression of miR-216a-3p in ESCC cells. Functionally, inhibition of miR-216a-3p reversed the effects of SNHG1 knockdown on ESCC development. These findings indicate that SNHG1 could sponge miR-216a-3p to modulate ESCC progression, similar to the previous study 40.

TMBIM6, known as BAX inhibitor 1 27, functions as an anti-apoptotic factor and is notably upregulated in various diseases, including breast cancer 44, laryngeal squamous cell carcinoma 45, and glioblastoma multiforme 46. In this study, we identified a shared binding site for miR-216a-3p in both TMBIM6 and SNHG1 using online tools. Based on this, we hypothesize that TMBIM6 may be involved in the ceRNA network regulated by SNHG1. Our study demonstrated a significant reduction in both the mRNA and protein levels of TMBIM6 in ESCC cells upon SNHG1 knockdown, which was reversed by the miR-216a-3p inhibitor, thereby confirming the presence of the SNHG1/miR-216a-3p/TMBIM6 axis. Calpain and Caspase-12 have been implicated in the modulation of cell proliferation and apoptosis, with TMBIM6 as a negative regulator of these processes 47, 48. Consistently, we observed that the SNHG1/miR-216a-3p/TMBIM6 axis regulated ESCC progression by modulating the expression of calpain and caspase-12. Finally, rescue experiments indicated that SNHG1 induced tumorigenic behavior by regulating the miR-216a-3p/TMBIM6 axis in ESCC. However, future investigations are warranted to elucidate the molecular mechanism through which TMBIM6 modulates genes involved in ESCC development.

This study has some limitations. For example, we did not use normal and other cancer cells as internal and external controls. Further studies are warranted.

In conclusion, our findings demonstrate that SNHG1 functions as an oncogenic lncRNA that promotes ESCC progression and is associated with adverse prognosis in ESCC patients. The SNHG1/miR-216a-3p/TMBIM6 axis holds promise as diagnostic and prognostic biomarkers, as well as therapeutic targets, for ESCC patients.

## Supplementary Material

Supplementary table.

## Figures and Tables

**Table 1:** Primer sequences for qRT-PCR.

Name	Primer	Sequence	Size
GAPDH	Forward	TCAAGAAGGTGGTGAAGCAGG	115bp
	Reverse	TCAAAGGTGGAGGAGTGGGT	
SNHG1	Forward	CTGTGTTCACTCCAGGCTGA	224bp
	Reverse	ACAGCAAACCCTCAACTGCT	
TMBIM6	Forward	TACCTTTGGGCAGAGTGGAG	245bp
	Reverse	TCAATATCAGGGAGCCCAAG	
U6	Forward	CGCTTCGGCAGCACATATAC	
	Reverse	AAATATGGAACGCTTCACGA	
miR-216a-3p	Loop primer	GTCGTATCCAGTGCAGGGTCCGAGGTATTCGCACTGGATACGACATAATCCCA	
	Forward	TGCGCTCACAGTGGTCTCTG	

**Table 2:** Association of SNHG1 expression with clinicopathological features of 50 cases of ESCC patients.

Clinicopathological features	Number of patients	SNHG1 expression		*P* value
		Low (n=28)	High (n=22)	
Age (years)				
≤60	21	13	8	0.474
>60	29	15	14	
Gender				
Male	40	25	15	0.064
Female	10	3	7	
Tumor volume, %				
<30	44	24	20	0.575
≥30	6	4	2	
Lymph node metastasis				
Negative	22	16	6	0.035*
Positive	28	12	16	
Depth of invasion				
Tis, T1	2	1	1	0.026*
T2	22	17	5	
T3	26	10	16	
Histological subtypes				
Well	3	3	0	0.047*
Moderate	23	9	14	
Poor	24	16	8	
Tumor status				
T1-T2	15	9	6	0.709
T3-T4	35	19	16	
TNM stage				
Ⅰ/Ⅱ	27	19	8	0.027*
Ⅲ	23	9	14	
