# Supplementary material for: LncRNA SNHG1 acts as a ceRNA for miR-216a-3p to regulate TMBIM6 expression in esophageal squamous cell carcinoma
Source: J Cancer. 2024 Apr 8;15(10):3128–39. doi: 10.7150/jca.95127 (PMC11064271; doi:10.7150/jca.95127)
Supplement: Supplementary file 1 — Supplementary table. [file jcav15p3128s1.pdf]

Supplementary Material

Sequence report - P202307130031

Product Number	Pseudolaric acid	target sequence
lnc3151124050308	RiboTM h-SNHG1 Smart Silencer	CCAGCATCTCATAATCTAT
		GTGAAGGAATGGGACAAGAC
		CCCTTGAGGACTGGCTGTCA
		AGCTGAGAGGTACTACTAAC
		GAGGACATCAGAAGGTGAA
		GCCAGCACCTTCTCTCTAA
